# Supplementary material for: Pathophysiological Significance of Neutrophilic Transfer RNA-Derived Small RNAs in Asymptomatic Moyamoya Disease
Source: Cells. 2021 May 1;10(5):1086. doi: 10.3390/cells10051086 (PMC8147334; doi:10.3390/cells10051086)

2D Structure	Local AU	Position

```
972                                   985
                    8mer-1a
5 ' - c t A G A G T C A C A G G a - 3 ' utr
          | : | | | | | | | | |
3 ' - g u U U U C A G U G U C C g - 5 ' tRF
 16 15   14 13      7 6 5 4 3 2
  3' Pairing          Seed
```

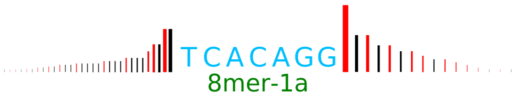

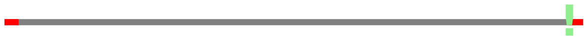

Supplement: Supplementary file 1 [file cells-10-01086-s001.zip › cells-1149161 3rd supplementary materials/Data Supplements/Data Supplement V.zip!/Data supplement 6-mRNAs targets of tsRNAs/tsRNA-Arg-CCT-008/Diagrams/tRF-Arg-CCT-008_vs_FTMT27400001780(CATG00000040931.1).pdf]
